# Converging prefrontal pathways support associative and perceptual features of conditioned stimuli

**DOI:** 10.1038/ncomms11546

**Published:** 2016-05-04

**Authors:** James D. Howard, Thorsten Kahnt, Jay A. Gottfried

**Affiliations:** 1 Department of Neurology, Feinberg School of Medicine, Northwestern University, Chicago, Illinois 60611, USA

## Abstract

Perceptually similar stimuli often predict vastly different outcomes, requiring the brain to maintain specific associations in the face of potential ambiguity. This could be achieved either through local changes in stimulus representations, or through modulation of functional connections between stimulus-coding and outcome-coding regions. Here we test these competing hypotheses using classical conditioning of perceptually similar odours in the context of human fMRI. Pattern-based analyses of odour-evoked fMRI activity reveal that odour category, identity and value are coded in piriform (PC), orbitofrontal (OFC) and ventromedial prefrontal (vmPFC) cortices, respectively. However, we observe no learning-related reorganization of category or identity representations. Instead, changes in connectivity between vmPFC and OFC are correlated with learning-related changes in value, whereas connectivity changes between vmPFC and PC predict changes in perceived odour similarity. These results demonstrate that dissociable neural pathways support associative and perceptual representations of sensory stimuli.

## Abstract (web summary)

Animals often need to form specific associations between perceptually similar stimuli and the different outcomes they may predict. Howard *et al*. find that the human brain accomplishes this via enhanced coupling between stable codes of sensory features and flexible codes of stimulus reward value.

## Graphical abstract

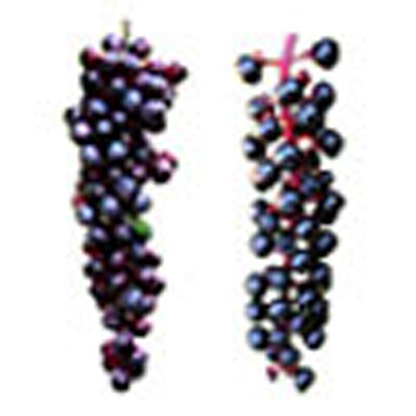

Foraging animals are faced with the dual challenge of generating stable representations of the outside world that can be sorted into categories based on shared perceptual features^1^, while at the same time remaining flexible enough to form specific associations between perceptually overlapping stimuli and different outcomes they may predict^2^. For example, we can describe the clusters of fruit-like objects dangling from both a grape vine and a pokeberry plant as ‘shiny' and ‘purple', and confidently classify them as berries. However, despite these obvious perceptual similarities, we can also readily learn that whereas the former promises a sweet, energy-rich source of food, the latter portends extreme gastrointestinal distress, or worse.

While forming associations between these berries and their two markedly different outcomes is of clear ecological importance, it is equally critical that these objects remain recognizable as shiny, purple berries so that future encounters with similar objects can be classified appropriately. A number of studies have explored how the mammalian brain forms and maintains perceptual categories^3^,^4^,^5^. In addition, there has been a substantial amount of research focused on how predictive reward signals arise by means of associative learning, and how these signals inform adaptive behaviour^6^,^7^,^8^,^9^,^10^,^11^,^12^. However, the mechanisms by which the human brain simultaneously encodes similarity-based perceptual categories while flexibly forming predictive associations remain largely unknown.

In principle, two potential mechanisms could underpin the formation of specific associations between sensory and reward representations. First, specific associations could be formed by restructuring, or ‘updating', categorical representations in object-level sensory cortices to reflect the newly acquired reward value. Modulation of sensory representations after both appetitive and aversive learning has been shown in virtually all sensory systems^13^,^14^,^15^,^16^,^17^,^18^,^19^, and is thought to play a major role in enhancing discrimination of behaviourally relevant stimulus features. Alternatively, categorical sensory representations themselves may remain intact, and instead, reward associations could be encoded via changes in connectivity between stimulus representations and higher-order reward-coding regions. To test these competing hypotheses in humans, we devised an olfactory functional magnetic resonance imaging (fMRI) paradigm of appetitive associative learning. Olfaction is a particularly advantageous modality through which to probe the associative flexibility of stimulus representations. Specifically, piriform cortex (PC) is unique among other sensory cortices in that it is directly and reciprocally connected with limbic and reward-based substrates such as the orbitofrontal cortex (OFC), ventromedial prefrontal cortex (vmPFC) and amygdala^20^.

In a pre-scanning behavioural testing session, we first established a set of four odour stimuli for each participant individually. Odours were selected such that they were matched in rated pleasantness, and conformed to a two-category perceptual similarity space comprising two minty odours and two citrus odours (Supplementary Fig. 1). In the main experiment, we acquired odour-evoked fMRI data, as well as behavioural ratings of odour pleasantness and similarity. This was done immediately before and after participants underwent appetitive classical conditioning using the four selected odours as conditioned stimuli (CS) and monetary reward as unconditioned stimuli (US) (Fig. 1a–c). Critically, one minty odour and one citrus odour were randomly selected to be paired with a $1.00 reward (mCS^+^ and cCS^+^), while the remaining minty odour and citrus odour were paired with no reward (mCS^−^ and cCS^−^) (Fig. 1b). This experimental design thus imposed a dual challenge: subjects must learn to flexibly acquire new information, in that only one odour from each category—but not its perceptually similar counterpart—is associated with the $1.00 reward, while maintaining stable representations of the odours' minty and citrus qualities.

On the basis of previous studies of odour coding in both humans and non-human model species^21^,^22^,^23^, we expected to find representations of the perceptual category of the odours in PC. We also expected to find representations of the acquired reward value of the odours in vmPFC, a region extensively implicated in value-based signalling and decision-making^24^,^25^,^26^. Here by implementing a combination of multivariate pattern analyses and connectivity techniques, we could directly test whether putative category-based odour representations become updated after learning to incorporate information about the associated reward value, or whether the learning-induced value changes are embodied in enhanced functional coupling between sensory and reward regions. Our findings provide evidence for the latter mechanism: whereas stable representations of odour identity and category were identified in OFC and PC, respectively, changes in OFC–vmPFC connectivity were predictive of learning-related changes in odour value, and changes in PC–vmPFC connectivity were predictive of changes in odour perceptual similarity. Together, these findings provide novel evidence for a sensory-reward network that can account for newly acquired associative value information while maintaining stable representations of CS.

## Results

### Learning-related behavioural effects

**Figure 1:**
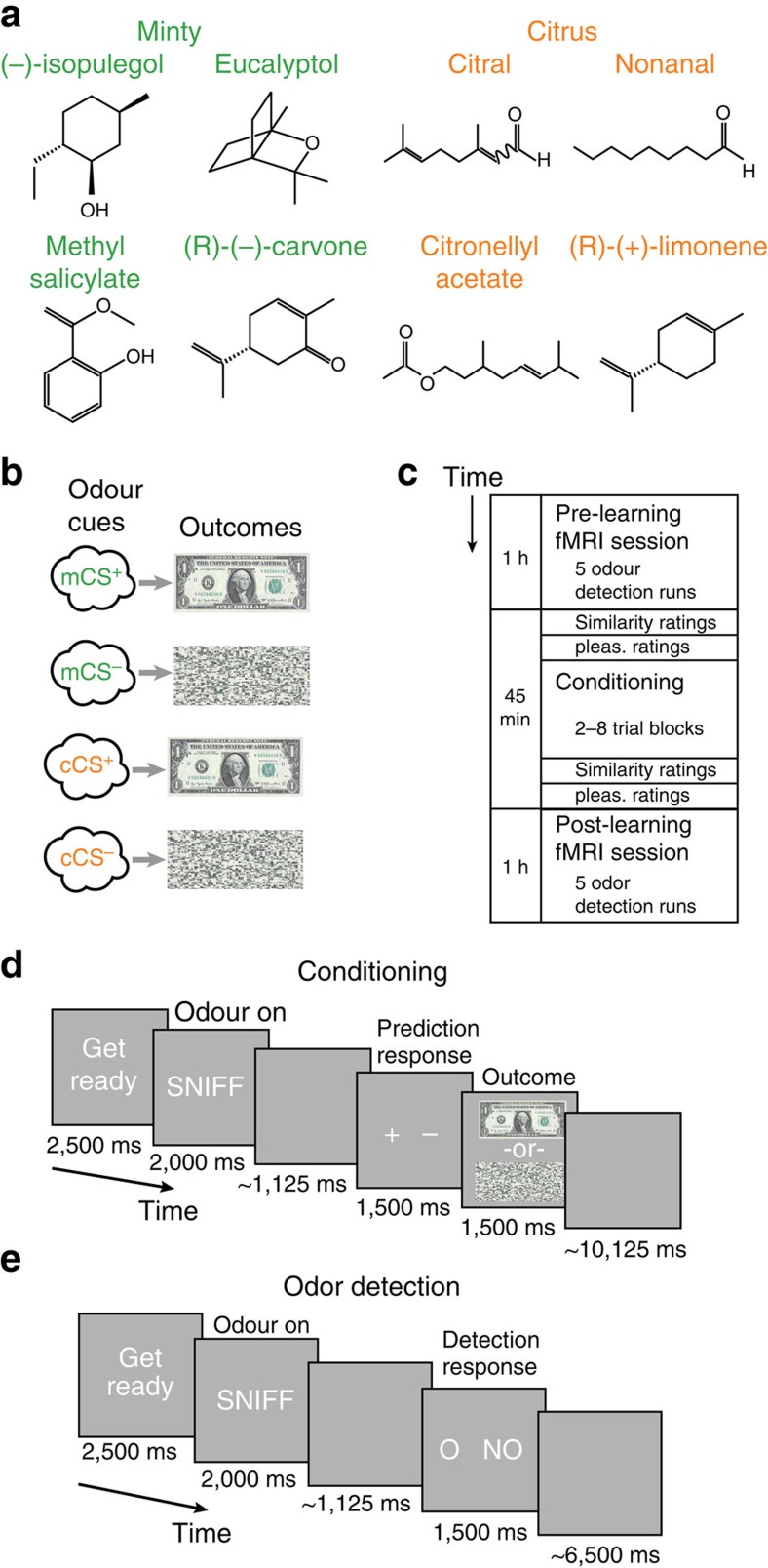
Stimuli and experimental paradigm. (**a**) Molecular diagrams of the eight odorants used in this experiment. Four of the odours are generally perceived as having minty qualities and four as having citrus qualities. (**b**) On the basis of ratings provided in an initial screening session (see online Methods and Supplementary Fig. 1), two minty odours and two citrus odours were selected that were matched as closely as possible in perceived pleasantness. One minty odour and one citrus odour were randomly chosen to be paired with $1.00 rewards (CS^+^), and the remaining two were paired with scrambled images (no reward, CS^−^). (**c**) Experimental timeline. The conditioning session was conducted outside the scanner in an adjacent testing room. (**d**) Conditioning trial timeline. Subjects were cued to sniff and presented with one of the four odour CS's, and after a jittered delay, were prompted to make an outcome prediction response. Following the response, subjects viewed either a picture of a $1.00 bill (signifying reward) or a scrambled image (signifying no reward). (**e**) Odour detection trial timeline. Subjects were cued to sniff and presented with either one of the four odour CS's or odourless air. After a jittered delay, subjects were prompted to respond as to the presence (O) or absence (NO) of odour.

**Figure 2:**
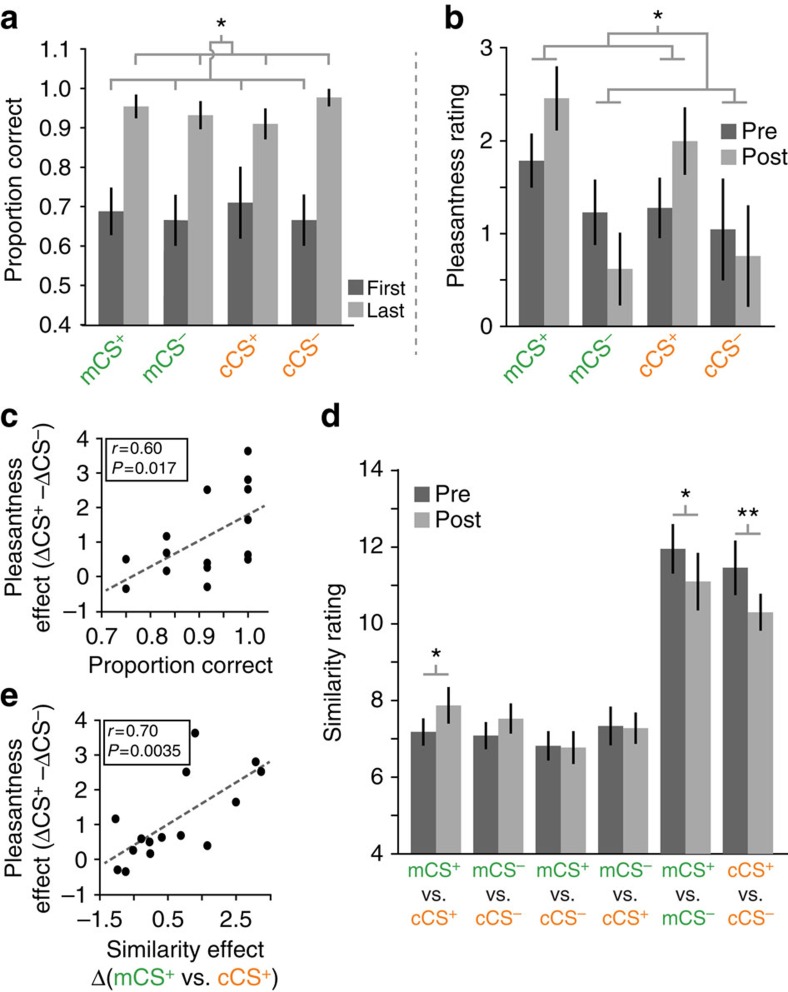
Reward prediction accuracy and behavioural ratings. (**a**) Proportion of correct responses for each of the four stimuli significantly increased from the first to the last block of conditioning. All subjects underwent at least two 12-trial blocks of the conditioning task. *Main effect of block, *P*<0.05. (**b**) Pre- and post-conditioning pleasantness ratings of the four odour CS's. Pleasantness was matched in the pre-conditioning session, but increased after conditioning for the two CS^+^'s. *Session-by-reward interaction, *P*<0.05. (**c**) Across subjects, the proportion of correct responses in the final block of the conditioning session predicted the session-by-reward interaction effect observed for the pleasantness ratings depicted in **b**. (**d**) Ratings of qualitative similarity between all possible odour pairs reveal that within-category similarity (mCS^+^ versus (vs.) mCS^−^, and cCS^+^ versus cCS^−^-) was significantly greater than across-category similarity in both rating sessions. The CS^+^'s became more similar to each other after conditioning, and the within-category CS^+^/CS^−^ pairs became less similar. *P<0.05, one-tailed paired *t*-test. **P<0.05, two-tailed paired *t*-test. (**e**) Across subjects, the increase in similarity for CS^+^ rating pairs predicted the same session-by-reward interaction in pleasantness depicted in **b**. Error bars represent within-subject s.e.m.

Subjects (*N*=15) underwent a classical conditioning session conducted in a separate testing room outside the fMRI scanner (Fig. 1c). To track learning, outcome prediction responses were made on each trial of this session (Fig. 1d). Conditioning proceeded in blocks of 12 trials until subjects either achieved 11/12 correct in a block, or completed 8 total blocks (average number of blocks completed=6.00±0.52, s.e.m.; Methods). Prediction response accuracy was significantly greater in the last block than in the first block (*F*_1,14_=41.1, *P*<0.001, main effect of block, repeated measures analysis of variance (ANOVA), *N*=15; Fig. 2a) and was above chance for each of the four conditions in the last block (*P*'s<0.001, *post hoc t*-tests), indicating that the subjects learned the associations between all odours and their outcomes.

We analysed the behavioural ratings provided before and after the conditioning session to test whether associative learning altered either the pleasantness (that is, value) or the perceptual similarity space occupied by the odour stimuli. Pre-conditioning pleasantness ratings were well matched amongst the odour cues (main effect of category, *F*_1,14_=0.69, *P*=0.42; main effect of reward outcome, *F*_1,14_=0.71, *P*=0.41; category × reward interaction, *F*_1,14_=0.12, *P*=0.73; repeated measures ANOVA, *N*=15). However, when comparing post-condition to pre-conditioning ratings we found a significant interaction between reward (CS^+^/CS^−^) and session (pre-/post-conditioning, *F*_1,14_=13.2, *P*=0.003, repeated measures ANOVA, *N*=15), indicating an increase in pleasantness for the CS^+^ (*post hoc* paired *t*-test, mCS^+^: *t*_14_=3.48, *P*=0.004; cCS^+^: *t*_14_=2.28, *P*=0.038) and a decrease in pleasantness for the mCS^−^ (*t*_14_=2.99, *P*=0.01; cCS^−^ was not significant: *t*_14_=1.03, *P*=0.32; Fig. 2b). Moreover, across subjects, the increase in odour pleasantness was directly related to the efficacy of associative learning, as indicated by a significant correlation between outcome prediction accuracy (in the final conditioning block) and the session-by-reward interaction in the pleasantness ratings (*r*=0.60, *P*=0.017, Pearson correlation, *N*=15; Fig. 2c). These results demonstrate that we successfully manipulated the value of the odours, such that CS^+^ odours increased in both absolute terms, and relative to CS^−^ odours.

Ratings of pairwise odour similarity indicated that, as expected, within-category pairs were significantly more similar than across-category pairs in both the pre- and post-conditioning rating sessions (*P*'s<0.05, paired *t*-tests on within-category ratings versus across-category ratings, for pre- and post conditioning separately; Fig. 2d). Thus, the odours were perceived as belonging to two distinct perceptual categories both before and after conditioning. We did, however, observe that the two rewarded odours (mCS^+^/cCS^+^) were rated as significantly more similar to each other after conditioning (paired *t*-test, *t*_14_=1.89, *P*=0.040, one-tailed; Fig. 2d), and the magnitude of this effect was correlated with the increase in pleasantness reported above (*r*=0.70, *P*=0.004, Pearson correlation, *N*=15; Fig. 2e). These findings suggest that although the mCS^+^ and cCS^+^ odours were perceived as belonging to different perceptual categories, pairing them with the same reward outcome increased the perceptual similarity between them. We also found a significant decrease in similarity for both the minty and citrus within-category pairs (mCS^+^/mCS^−^, *t*_14_=1.87, *P*=0.047, one-tailed; cCS^+^/cCS^−^, *t*_14_=2.83, *P*=0.013, paired *t*-tests, *N*=15; Fig. 2d), though these changes were not correlated with any measures of learning or pleasantness.

Participants performed an odour detection task during the fMRI scanning sessions, which were conducted immediately before and after conditioning. On each trial of this task, participants were cued to sniff either one of the four odour stimuli or odourless air, and then asked to indicate whether or not an odour was present (Fig. 1e). We found no significant main effects or interactions when analysing the proportion of correct responses in this task in a three-way ANOVA with reward, session and category as factors (*P*'s>0.27, Supplementary Fig. 2). We did find a main effect of category on reaction times (*F*_1,14_=10.2, *P*=0.0065, repeated measures ANVOA, *N*=15), but no other main effects or interactions (Supplementary Fig. 2). Taken together, these null findings suggest that the odour-evoked activity collected during the detection task scans was unlikely to reflect differences in response confidence or other performance-based measures that might have complicated interpretation of the imaging data.

### Predicted value is coded in vmPFC

**Figure 3:**
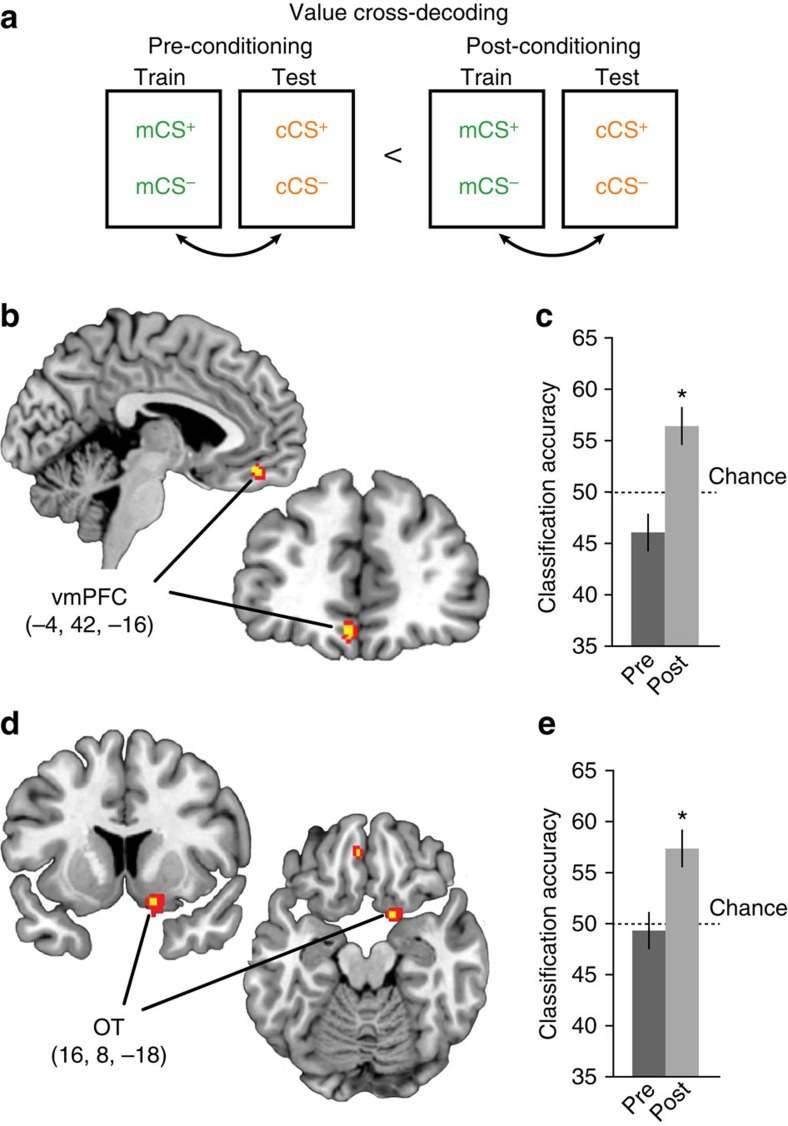
Searchlight decoding analysis for representations of associative value. (**a**) Value-based cross-decoding schematic. Patterns of fMRI activity within each searchlight sphere evoked by CS^+^ and CS of one category were used to train the classifier, which was then tested on CS^+^ and CS^−^ patterns of the other category. Post-conditioning accuracies were compared with pre-conditioning accuracies at the group level with paired *t*-tests. (**b**) Value representations emerged after conditioning in left vmPFC, which were significantly above chance (**c**) in the post-conditioning session. (**d**) Expected value was also represented in right OT, which also demonstrated above-chance classification in the post-conditioning data alone (**e**). Error bars represent between-subject s.e.m. *P<0.05, paired *t*-test against chance. Brain activations in **b** and **d** displayed at *P*<0.001 uncorrected (red) and *P*<0.05 small-volume corrected for family-wise error (yellow). Coordinates in **b** and **d** refer to voxel of peak decoding accuracy.

The behavioural findings described above demonstrate that subjects formed associations between the monetary outcomes and the specific odours that predicted them. To identify the neural changes that accompany the formation of these CS-specific reward associations, we analysed the odour-evoked fMRI data from the odour detection task, conducted before and after the conditioning session. Previous studies have shown that reward value is encoded in distributed patterns of activity in the prefrontal cortex^27^,^28^. To optimize sensitivity to such distributed fMRI representations, we implemented a support vector machine pattern classification analysis in a searchlight-based manner^29^, which provides an unbiased test of information content at each imaged section of the brain (Methods). Specifically, we tested whether the observed increase in pleasantness ratings after learning for the CS^+^ odours was mirrored in changes in odour-evoked fMRI patterns. For this, the classification analysis was performed separately on imaging data from the pre- and post-conditioning sessions at the single-subject level, and then tested at the group level for session-related effects (Methods). To ensure that above-chance decoding in this analysis could only be driven by the predicted value of the CS, independent of any categorical or stimulus-specific information, we implemented a cross-classification technique wherein the classifier was trained to discriminate fMRI patterns evoked by CS^+^ versus CS^−^ of a given perceptual category (mint or citrus), and then tested on CS^+^ versus CS^−^ patterns evoked by the other category, and vice versa (Fig. 3a).

Using this approach, we found significantly higher value-based classification accuracy in post- versus pre-conditioning data in vmPFC (peak voxel: *x*=−4, *y*=42, *z*=−16, *t*_14_=6.12, *P*_FWE,SVC_=0.0029, paired *t*-test, *N*=15; Fig. 3b). *Post hoc* one-sample *t*-tests (all *N*=15) confirmed that post-conditioning accuracy was significantly above chance in vmPFC (*t*_14_=3.49, *P*=0.004, 95% confidence interval (CI) (52.50–60.46)) (Fig. 3c), but did not differ from chance in the pre-conditioning data (*t*_14_=−1.91, *P*=0.08, 95% CI (42.41–50.44)). In addition, we found value representations in the olfactory tubercle (OT; *x*=16, *y*=8, z=−18, *t*_14_=4.29, *P*_FWE,SVC_=0.036; Fig. 3d), which also showed above-chance accuracy in post conditioning (*t*_14_=4.76, *P*<0.001, 95% CI (54.07–60.75)), but not in pre-conditioning (*t*_14_=0.38, *P*=0.71, 95% CI (45.71–52.99); Fig. 3e). These findings indicate that the predicted reward value of the odour CS was encoded in vmPFC and OT.

The identification of pattern-based value signals does not necessarily preclude the possibility that value signals might also be reflected in global fMRI signal changes. To test for this possibility, we conducted a univariate analysis using a more traditional general linear model (GLM) approach on spatially smoothed functional images. Using a session-by-reward contrast at the group level we found no regions that exhibited greater activity to the CS^+^ odour cues relative to CS^−^ after learning, even at a liberally thresholded level of *P*<0.05, uncorrected for multiple comparisons. These null results underscore the utility of multivariate methods for uncovering information content contained within distributed patterns of neural activity.

### Perceptual category is coded in PC

**Figure 4:**
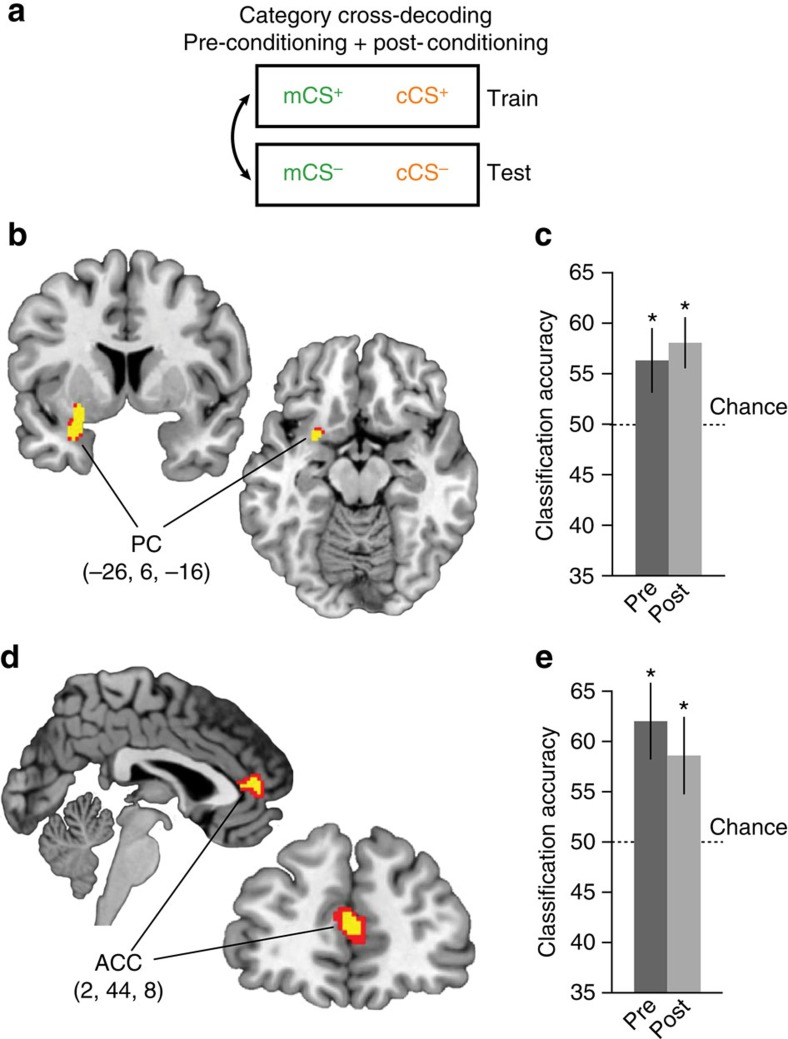
Searchlight decoding analysis for representations of perceptual category. (**a**) Category-based cross-decoding schematic. Across-category patterns of fMRI activity evoked by CS's of the same reward level were used to train the classifier, which was then tested on across-category patterns evoked from the opposite reward level (and vice versa). Classification accuracy was tested on combined data from pre-conditioning and post-conditioning sessions, and accuracy was tested at the group level in a one-sample *t*-test. (**b**) Category representations were found in left PC, a region previously associated with odour category coding, with above-chance classification (**c**) in both pre- and post-conditioning sessions. (**d**) Category information was also represented in anterior cingulate cortex (ACC), which demonstrated above-chance classification in both pre- and post-conditioning sessions (**e**). Error bars represent between-subject s.e.m. *P<0.05, paired *t*-test against chance. Brain activations in **b** and **d** displayed at *P*<0.001 uncorrected (red) and *P*<0.05 small-volume corrected for family-wise error (yellow). Coordinates in **b** and **d** refer to voxel of peak decoding accuracy.

Similarity ratings indicated that although the two rewarded odours became more similar to each other after learning, the categorical perceptual structure of the odours was preserved. We therefore tested for regions that encoded the perceptual category of the odours using data from both pre- and post-conditioning fMRI sessions combined. To assess olfactory categorical coding, we again utilized multivariate fMRI analysis methods, based on extensive prior work indicating that odour information takes the form of sparsely distributed and overlapping patterns of ensemble activity in olfactory cortex^21^,^22^,^30^. As such, multivariate pattern analysis (MVPA) methods are ideally suited for characterizing these representations. For this analysis, the classifier was trained on patterns evoked by mCS^+^ versus cCS^+^, and tested on mCS^−^ versus cCS^−^ (and vice versa) (Fig. 4a). By training and testing within-reward level, we ensured that above-chance classification could not be confounded by differences in predicted value. Moreover, training and testing on different odours from the same perceptual category ensured that decoding accuracy truly reflected category as opposed to stimulus-identity information.

We found robust category coding in PC (*x*=−26, *y*=6, *z*=−16, *t*_14_=4.60, *P*_FWE,SVC_=0.007; Fig. 4b). *Post hoc* one-sample *t*-tests (all *N*=15) revealed that classification in PC was significantly above chance in the pre- and post-conditioning sessions separately (pre-conditioning, *t*_14_=1.98, *P*=0.034, one-tailed, 95% CI (49.49–63.24); post conditioning, *t*_14_=3.20, *P*=0.006, 95% CI (52.68–63.54); Fig. 4c), without any difference between pre- and post-conditioning accuracy (*t*_14_=0.52, *P*=0.61), suggesting that category coding in this region was not altered by appetitive learning. Interestingly, we found a similar effect in the anterior cingulate cortex (*x*=2, *y*=44, *z*=8, *t*_14_=4.60, *P*_FWE,SVC_=0.032; Fig. 4d), which also exhibited significant above-chance classification in both sessions separately (pre-conditioning, *t*_14_=3.16, *P*=0.007, 95% CI (53.89–70.30); post conditioning, *t*_14_=2.24, *P*=0.042, 95% CI (50.38–66.93); pre- versus post conditioning, *t*_14_=0.82, *P*=0.42) (Fig. 4e).

To test for regions in which category coding changed by associative learning, we compared post-conditioning with pre-conditioning classification accuracy at the group level, as was done for the value-based decoder above. This analysis identified no regions that exhibited a significant learning-related change in category coding, suggesting that stimulus–reward associations are unlikely to be based on category representations in this experimental context.

### Stimulus identity is coded in posterior OFC

**Figure 5:**
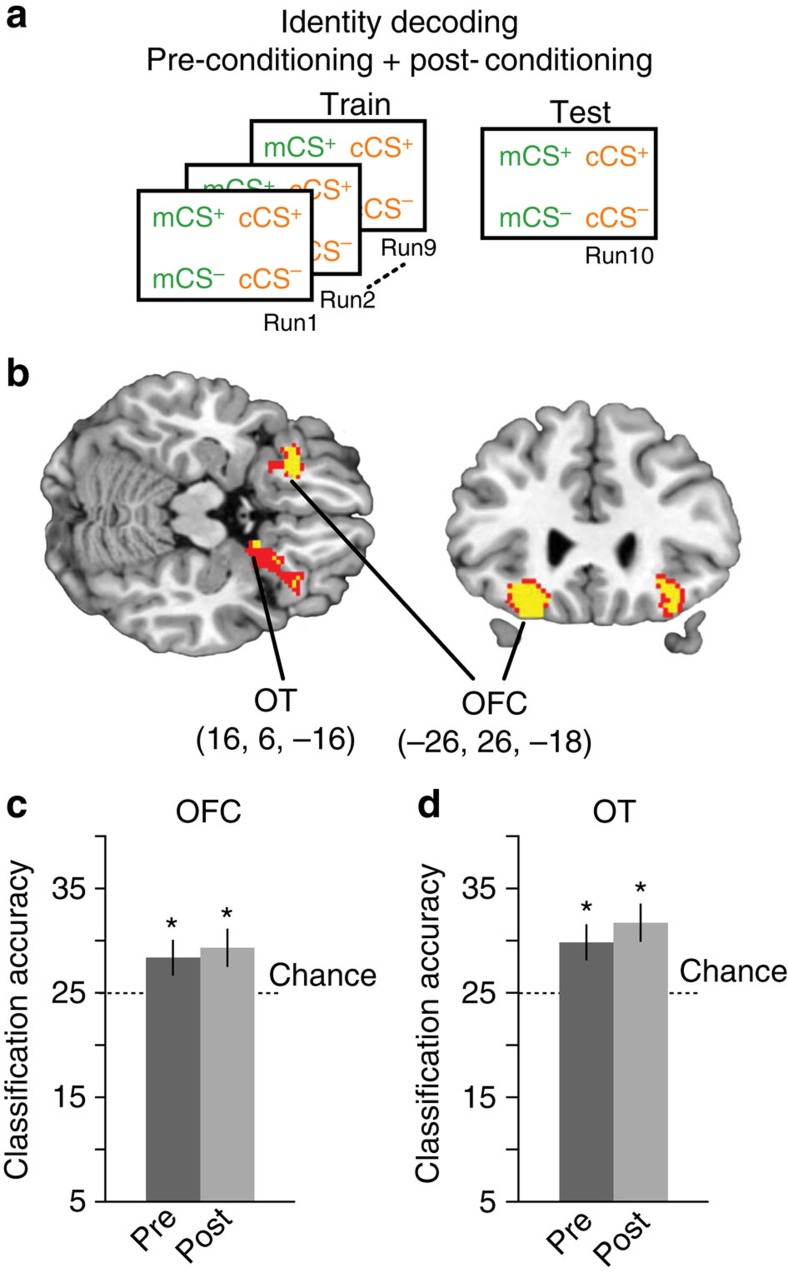
Searchlight decoding analysis for representations of stimulus identity. (**a**) Identity-based decoding schematic. The classifier was trained and tested on patterns evoked by all four CS's. (**b**) Stimulus identity representations were found in left posterior OFC and right OT. Identity-based classification in both (**c**) left posterior OFC and (**d**) right OT was significantly above chance in both pre- and post-conditioning sessions. Error bars represent between-subject s.e.m. *P<0.05, paired *t*-test against chance. Brain activations in **b** displayed at *P*<0.001 uncorrected (red) and *P*<0.05 small-volume corrected for family-wise error (yellow). Coordinates in **b** and **d** refer to voxel of peak decoding accuracy.

As discussed above, learning did not affect representations of perceived odour category in the brain. We therefore reasoned that non-categorical, identity-based representations of the odour stimuli might have exhibited modulation to reflect the newly acquired stimulus–reward associations. To test this idea, we trained the classifier simultaneously on patterns evoked by all four CS's (four-way classification) in a subset of fMRI runs, and then tested on patterns from the left-out run (Fig. 5a and Methods). This analysis therefore tests for discriminable patterns of activity evoked by each of the four odours with no requisite value-based or categorical organization.

We found no regions that exhibited significantly different identity coding across imaging sessions. However, when combining data from both sessions, we found significant above-chance classification in left posterior OFC (*x*=−26, *y*=26, *z*=−18, *t*_14_=6.56, *P*_FWE,SVC_=0.007) and in the right OT extending towards posterior OFC (*x*=16, *y*=6, *z*=−16, *t*_14_=6.04, *P*_FWE,SVC_=0.012; Fig. 5b). *Post hoc* one-sample *t*-tests (all *N*=15) confirmed that identity-based classification was significantly above chance in the pre- and post-conditioning sessions separately for both OFC (pre-conditioning, *t*_14_=3.58, *P*=0.003, 95% CI (24.73–32.13); post conditioning, *t*_14_=4.01, *P*=0.001, 95% CI (25.44–33.32); Fig. 5c) and OT (pre-conditioning, *t*_14_=2.77, *P*=0.015, 95% CI (26.10–33.69); post conditioning, *t*_14_=3.74, *P*=0.002, 95% CI (28.15–35.40); Fig. 5d). In another *post hoc* analysis, we confirmed that the identity coding observed in these regions was not driven by discrimination between any particular pair of odours. For this, we trained and tested a classifier on all possible pairs of odour stimuli separately in the pre- and post-conditioning sessions. Classification accuracy was significantly above chance for all possible odour pairs in both scanning sessions in OFC (*P*'s<0.05, one-sample *t*-tests, *N*=15), confirming that odour identity information in this region was stable across stimuli and sessions. We found a similar result in OT, with the exception that one of the within-category pairs was at chance in the pre-conditioning session, but above chance in post. The profile in OT is compatible with the fact that both value-based and identity-based information were independently identified in this region.

### Dissociable pathways mediate distinct behavioural changes

**Figure 6:**
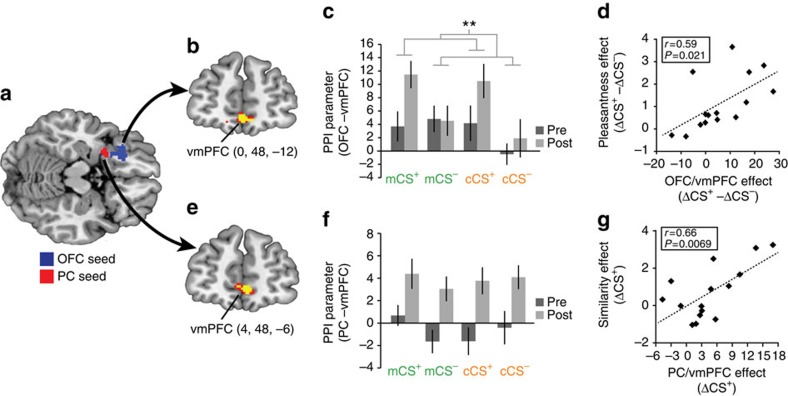
Connectivity analysis with OFC and PC as seed regions. (**a**) Seed regions for the PPI connectivity analysis were selected as clusters of activation from the category cross-decoding (PC) and stimulus identity decoding (OFC) analysis, thresholded at *P*<0.001 uncorrected. (**b**) Connectivity between the OFC seed region and vmPFC was enhanced after learning. (**c**) PPI parameters at the peak voxel in the OFC-coupled vmPFC cluster corresponding to each CS and session. *Post hoc* three-way ANOVA revealed a session-by-reward interaction in this region. (**d**) OFC/VMPFC connectivity session-by-reward interaction effect predicted session-by-reward pleasantness rating effect. (**e**) Connectivity between the PPC seed region and vmPFC was enhanced after learning. (**f**) PPI parameters at the peak voxel in the PC-coupled vmPFC cluster corresponding to each CS and session. (**g**) PC/vmPFC connectivity parameter change for CS^+^ odours predicted similarity rating change for CS^+^ odours. Error bars represent within-subject s.e.m. **P<0.05, interaction in a three-way repeated measures ANOVA. Brain activations in **b** and **e** displayed at *P*<0.001 uncorrected (red) and *P*<0.05 small-volume corrected for family-wise error (yellow). Coordinates in **b** and **d** refer to voxel of peak connectivity effect.

We found evidence for odour category and odour identity representations most prominently in left PC and left posterior OFC, respectively. Decoding accuracy in both of these regions was stable across learning sessions, suggesting that reward learning did not induce a profound modulation of these sensory representations. However, we reasoned that the observed learning-related changes in behaviour might instead be explained by altered connectivity between either of these regions and emerging reward representations in the vmPFC. To test this possibility, we implemented two independent connectivity analyses using the generalized psychophysiological toolbox^31^: one with identity-coding OFC as a seed region, and one with category-coding PC as a seed region (Fig. 6a). In both analyses we first tested for brain regions that exhibited a general learning-related modulation of connectivity with the seed region (Methods), and then tested how these effects related to observed changes in behaviour.

In the OFC seed region analysis, we found enhanced connectivity after conditioning in vmPFC (*x*=0, *y*=48, *z*=−12, *t*_14_=4.91, *P*_FWE,SVC_<0.001, paired *t*-test, *N*=15; Fig. 6b), directly adjacent to the value-coding region described above (Fig. 3). A *post hoc* test for interactions on condition-specific psychophysiological interaction (PPI) parameters (extracted at the peak vmPFC voxel) revealed a session-by-reward interaction (*F*_1,14_=5.80, *P*=0.030, repeated measures ANOVA, *N*=15), suggesting that the main effect of session in this region was driven by an increase in connectivity for the CS^+^ odours after conditioning (Fig. 6c). Interestingly, this odour-specific increase in OFC–vmPFC connectivity was related to learning-related changes in odour pleasantness, as indicated by a significant correlation between odour-specific increases in connectivity and pleasantness ratings (*r*=0.59, *P*=0.021, Pearson correlation, *N*=15; Fig. 6d), but not with the increase in similarity ratings for the two rewarded odours (*r*=0.45, *P*=0.08, Pearson correlation, *N*=15). These findings suggest that functional coupling between odour identity codes in OFC and predictive value codes in vmPFC supports the odour-specific increase in value.

Even though category representations did not subserve the formation of odour-specific associations, we found a similar increase in connectivity with the vmPFC for the PC seed region (4, 48, −6, *t*_14_=4.24, *P*_FWE,SVC_=0.0093; Fig. 6e). However, a *post hoc* ANOVA revealed no significant interaction between session and reward (*F*_1,14_=0.001, *P*=0.98, repeated measures ANOVA, *N*=15), indicating that PC–vmPFC connectivity was nonspecifically enhanced for all four odours after conditioning (Fig. 6f). However, we found that the change in PC–vmPFC connectivity for CS^+^ odours was significantly correlated with the increase in similarity between the CS^+^ odours (*r*=0.66, *P*=0.007, Pearson correlation, *N*=15; Fig. 6g). These findings suggest that changes in perceived similarity imposed by a shared predicted outcome are driven by an increased coupling between reward-coding and category-coding regions, in the absence of changes in local similarity-based representations.

## Discussion

The ability to group sensory stimuli into categories based on shared perceptual features is a fundamental ability of the central nervous system. However, categorical perception can hinder associative learning in the case where closely related stimuli predict different, or even opposing, outcomes. Here we demonstrate that the human brain overcomes this challenge by maintaining two distinct stimulus representations, each subserving different functions. Perceptual category information was represented in PC, while odour identity was coded in OFC. Critically, learning-related changes in connectivity between identity-coding OFC and value-coding vmPFC predicted value-based behavioural changes. These findings highlight a neural mechanism by which the brain can form specific odour–reward associations in the face of perceptual ambiguity.

In our study, representations of predicted odour–reward value emerged in the vmPFC after appetitive Pavlovian conditioning. Previous studies have also linked this region to value coding in humans^24^,^26^, though these studies typically measured subjective value in the context of purchasing decisions^32^,^33^,^34^. Here implementation of an odour detection task during fMRI scanning did not require active valuation of the odours on the part of our participants, promoting the idea that vmPFC also participates in more automatic, stimulus-driven valuation^35^,^36^. Pavlovian value representations in prefrontal regions such as vmPFC have been most consistently revealed using pattern-based analyses, suggesting that these stimulus-driven signals are encoded in a distributed fashion in the vmPFC in the absence of global fMRI signal changes^28^,^35^,^37^.

Value representations were also found in the OT. Interestingly, this region also exhibited odour identity coding in both pre- and post-conditioning sessions. While these findings correspond to OT as defined in a neuroanatomical atlas of the human brain^38^, a consensus on the location of the OT in humans has not been reached^39^. However, given that OT is extensively connected with the ventral striatum^40^ and exhibits both reward-related^41^,^42^ and olfactory sensory functionality in rodent model systems^43^, it may constitute an interface between odour identity and reward value codes in the present study.

Somewhat surprisingly, we did not observe value representations emerging after learning in OFC, a region previously shown to code value in both animal^11^,^44^,^45^,^46^ and human studies^6^,^7^,^32^. One potential reason that we did not observe signals related to expected reward value in the present study relates to the modality of the reward, which in this case is money. Human studies using monetary reinforcers tend to show expected value signals in vmPFC as opposed to OFC^34^,^47^,^48^,^49^. These studies represent an emerging pattern of findings, in which stimuli and rewards that are more directly tied to sensory aspects are represented in central/posterior OFC, while more abstract rewards and associated decision variables are represented in medial OFC/vmPFC^26^,^33^,^37^,^50^,^51^,^52^. Moreover, neuroanatomical studies have identified two distinct networks in OFC based on patterns of connectivity: a ‘sensory' network that comprises much of the central/lateral and posterior OFC, and a ‘visceromotor' network that comprises more medial OFC regions, as well as vmPFC^53^. This anatomical distinction, coupled with the aforementioned empirical studies, may explain our finding that the sensory identity of our odour stimuli was found in posterior OFC, a region more intimately connected to olfactory sensory regions, while the more abstract associative value of these stimuli emerged in vmPFC after learning.

The learning task implemented here involved discrimination training between the two minty and two citrus odours. According to theories of stimulus generalization^54^,^55^, this training should have increased the perceived difference between the CS^+^ and CS^−^ within each category. In line with this prediction, similarity ratings of the two odours belonging to the same category (but predicting different outcomes) decreased after learning. In contrast, because odours from different categories predicted the same reward outcome, our task should have increased the functional equivalence of these odours^56^,^57^. Specifically, according to associative mediation theory^57^, equivalence is achieved through retrieval of a common association, or outcome, shared between two stimuli. We found evidence for emergent equivalence, in that the two rewarded odours, despite belonging to distinct perceptual categories, became perceptually more similar to each other after learning. Interestingly, the increase in similarity was directly related to connectivity changes between category-coding PC and value-coding vmPFC. This finding suggests that via mechanisms of acquired equivalence, the retrieval of a common value-based association between perceptually dissimilar odours can be implemented through a shared increase in connectivity between stimulus representations in PC and a common reward value representation in vmPFC.

We did not find a significant modulation of odour representations after conditioning in PC. This is in contrast to previous studies demonstrating this region's plasticity in response to a variety of learned associations and contexts^58^,^59^,^60^. We believe there are a few possible reasons why conditioning in this case did not have a detectable impact on odour coding in PC. First, it is important to note that even at baseline, it was very easy to distinguish odours belonging to the same category, and to distinguish odours belonging to different categories. This differs from our prior work demonstrating learning-related changes in PC and OFC, in which the odour stimuli were perceptually indistinguishable at baseline^18^. Therefore, in the present study, further perceptual changes in odour identity and category were likely to be modest at best (as suggested in Fig. 2d), reducing the chance of identifying significant coding changes over time. Second, this study is one of the few human olfactory studies using monetary reward as the US. Compared with our other conditioning studies using aversive shock as the US^18^,^61^, the use of an appetitive US may simply have elicited weaker changes in perceptual coding. Third, initial behavioural testing (occurring ∼7 days before scanning) involved presentation of 4 minty odours and 4 citrusy odours to optimize the final stimulus set. It is possible that categorical pre-exposure might have had the effect of anchoring subjects' percepts more tightly to the two odour categories, reducing the likelihood of inducing coding changes in PC or OFC. However, we acknowledge that associative learning still might have modulated odour representations in a more fine-grained manner at the level of single-unit firing activity, yet would have eluded detection using fMRI given that this technique samples the activity of thousands of neurons within a single voxel.

Whereas connectivity between vmPFC and categorical representations of the odour stimuli in PC predicted similarity-based behavioural changes, connectivity between vmPFC and non-categorical identity representations in OFC predicted value-based behavioural changes. The finding of odour identity representations in OFC aligns with a number of previous studies, including human fMRI (summarized in ref. 62) and single-unit recording experiments in both rodents^63^ and monkeys^64^. These studies demonstrated odour-induced OFC responses in the absence of value manipulations or cognitive judgments. However, the utility of such sensory representations in addition to those found in primary olfactory cortex had remained elusive, but becomes apparent in the context of our current findings. Specifically, the representation of odour identity codes in parallel with odour category codes offers an efficient mechanism to disambiguate similar stimuli while keeping perceptual structure anchored and intact.

Our results suggest that specific stimulus–outcome associations are formed based on stable identity representations in OFC through enhanced connectivity with reward-coding regions in the vmPFC. This mechanism can be described as an enhanced read-out of sensory information by a higher cognitive brain region to represent stimulus-specific outcome expectancies. Similar mechanisms have been suggested to mediate perceptual learning. More specifically, reinforcement learning-driven improvements in stimulus discriminability have been shown to correlate with signal changes in higher-order decision-related regions, while early sensory representations remain unaltered^65^,^66^. Our current findings extend this idea to the domain of reward learning by showing that changes in connectivity between sensory and higher cognitive regions are directly linked to the formation of specific stimulus–reward associations.

It is worth emphasizing that definitive interpretation of the connectivity findings as they relate to the pattern-based analyses remains difficult. Specifically, it is unclear precisely how the fMRI time courses forming the basis of our connectivity analyses—which are necessarily averaged across voxels within a particular region—relate to information contained within those same regions in distributed patterns of voxel activity. Thus, while the acquisition of value information induced by our learning task clearly modulated both pattern-based odour responses and connectivity, direct understanding of the relationship between the MVPA and connectivity findings remains elusive. Moreover, it is unclear whether the observed change in connectivity between OFC and vmPFC after learning is due to a modulation of the influence of OFC activity on vmPFC after learning, or a modulation of the psychological context that simultaneously modulates activity in both regions^67^.

Taken together, the findings presented here provide a mechanistic account for how identity-based and category-based odour representations interact with prefrontal brain regions to simultaneously support distinct reward-related changes in behaviour. While the connectivity analysis does not inform directionality, we speculate that the OFC–vmPFC connectivity change may represent a sensory-reward network coming ‘online' to access behaviourally relevant stimulus information that is disambiguated from potentially confounding perceptual similarity-based codes. Subsequently, the vmPFC–PC connectivity change may constitute a ‘top-down' effect whereby the common associated outcome imparts a newly acquired measure of shared information. That these behavioural changes were directly related to connectivity changes, as opposed to fluctuations in global signal or representational modulation, exemplifies a growing recognition in cognitive neuroscience that cooperative interactions between brain regions are critical for supporting complex behaviours^68^,^69^.

## Methods

### Participants

Seventeen right-handed, non-smoking participants (10 female, mean age=24.6±2.8 years) with no history of neurological disorders signed consent forms indicating their willingness to participate in this experiment according to the protocols approved by the Northwestern Institutional Review Board. Owing to excessive head motion during scanning, two participants were removed from all analyses, resulting in data from a total of 15 subjects reported here.

### Stimuli and delivery

Eight high-purity synthetic odorants (four minty: isopulegol, L-carvone, methyl salicylate, eucalyptol; four citrusy: citral, (R)-(+)-limonene, citronellyl acetate, nonanal; Fig. 1a) were obtained from Sigma Aldrich (St Louis, MO) and delivered directly to subjects' noses using a custom-built olfactometer. This system is capable of mixing odourized air, diverted via separate channels through the gaseous headspace of 20-ml amber bottles containing 1 ml of undiluted liquid odorant, with odourless air diverted through an empty amber bottle. To equalize perceived odour intensity as best as possible across the stimulus set, before all testing sessions we adjusted the ratio of odourized to odourless air individually for each odorant while maintaining a constant total flow rate of 3.2 l min^−1^ (see Supplementary Table 1 for the list of flow ratios (odourized to odourless air) used for each odorant). For all testing sessions odours were delivered using the olfactometer, with the exception of pairwise odour similarity rating sessions (see below), during which subjects sniffed as prompted from labelled amber bottles containing odorants diluted with diethyl phthalate at low concentrations (6–20%).

### fMRI data acquisition

Gradient-echo T2-weighted echoplanar images (EPIs) were acquired with a Siemens Trio 3T scanner using parallel imaging and a 32-channel head coil with the following parameters: repetition time=1.51 s; echo time=20 ms; matrix size=128 × 120 voxels; field-of-view=220 × 206 mm; in-plane resolution=1.72 × 1.72 mm; slice thickness=2 mm; gap=1 mm; acquisition angle=30° rostral to the intercommissural line; and slices per image=24. See Supplementary Fig. 3 for a depiction of the scanner coverage on a sample subject. We also acquired 10 whole-brain EPI volumes with the same scanning parameters as above except 48 slices per image to aid in the spatial normalization (see below), as well as a 1 mm^3^ isotropic T1-weighted structural scan for anatomical reference.

### Experiment paradigm: pre-scanning behavioural testing

In this testing session, conducted ∼7 days before the scanning session, subjects first rated the pleasantness of 8 odours in random order on an analogue scale from −10 (most disliked sensation imaginable) to +10 (most liked sensation imaginable). Each of the 8 odours was rated 3 times for a total of 24 ratings. On the basis of these ratings, 2 minty odours and 2 citrusy odours were selected such that variance in pleasantness across the 4 was minimal, and these 4 odours were used in all subsequent testing. Thus, the specific set of odours used in the main experiment varied from subject to subject (Supplementary Table 2). Subjects then rated the intensity (anchors ‘undetectable' and ‘strongest imaginable') and familiarity (anchors ‘least familiar imaginable' and ‘most familiar') 2 times for each of the 4 selected odours. Subjects then provided ratings of pairwise qualitative similarity (anchors ‘not alike at all' and ‘identical') 4 times for each of the 6 possible pairs of the selected odours, for a total of 24 similarity ratings.

### Experiment paradigm: main experiment

The main experiment was conducted in 3 phases: a pre-learning fMRI scanning session, a conditioning session and a post-learning fMRI scanning session (Fig. 1c). The pre-learning session consisted of 5 fMRI odour detection runs, each of which lasted 6.2 min and involved the acquisition of 245 functional EPI volumes. Each fMRI run consisted of 25 trials: 5 each of the 4 selected odours and odourless air, presented in random order. On each trial, subjects were presented with either one of the four odours or odourless air, and after a variable delay responded via mouse button press as to the presence (or absence) of odour (Fig. 1e). The post-learning scanning session was identical to the pre-learning session except that the stimulus order was independently randomized.

After the pre-learning fMRI session, subjects were removed from the scanner and taken to a separate testing room to complete the reward learning phase. Because the screening session was conducted ∼1 week in advance of the main experiment, we first re-established a pre-learning baseline for pleasantness (three repetitions per odour) and pairwise similarity ratings (two repetitions per odour pair). For the conditioning session, one minty and one citrusy odour were randomly chosen to be paired with monetary reward outcomes (CS^+^), while the remaining two odours were paired with no reward outcomes (CS^−^) with 100% contingency. On each trial of this session, subjects were presented with one of the four odours, and after a variable delay indicated via mouse button press whether that odour would lead to a reward (or no reward, Fig. 1d). Immediately after the prediction response was made, subjects were presented with either a picture of a $1 bill (reward) or a scrambled image of the bill (no reward). Before the learning session, subjects were instructed that each time they viewed a $1 bill, they in fact earned one dollar, and that all of the dollars they viewed would add up to an amount that they would receive at the end of the experiment. Subjects were also instructed that the responses they made did not affect the outcomes they viewed (that is, the outcome was tied to the odour, and not to the response), but they could earn extra money by making accurate prediction responses. Learning proceeded in blocks of 12 trials, after which subjects were given feedback as to the percentage of correct responses made in that block. Once prediction accuracy exceeded 90% in a block (11 out of 12 correct), the learning phase was terminated and subjects proceeded to make post-learning ratings of odour pleasantness and pairwise similarity. After a short break, subjects then returned to the scanner to complete the post-learning odour detection runs.

### Sniff recording and analysis

Sniffing activity data were acquired during the fMRI odour detection runs using MR-compatible piezoelectric resistive effort bands affixed around the chest and abdomen of each subject, and recorded using PowerLab equipment (ADInstruments, Dunedin, New Zealand) at a sampling rate of 1 kHz. Sniff traces for each fMRI run were temporally smoothed using a moving window spanning 500 ms and normalized by subtracting the mean and dividing by the s.d. across the entire run trace. The onset of inhalation was determined by finding the time of the minimum signal value within a window spanning 1 s on either side of the sniff cue presentation. These trial-by-trial sniff onset times were used as event onsets in the general linear models described below. For each trial, sniff peak amplitude, sniff duration and inhalation volume were calculated and sorted by trial type for subsequent analyses (Supplementary Fig. 4).

### Image preprocessing

To correct for head motion during scanning, for each subject all functional images across pre- and post-conditioning scanning sessions were aligned to the first acquired image using SPM8 (www.fil.ion.ucl.ac.uk/spm/). Spatial normalization was performed by first co-registering each subject's mean whole-brain EPI volume to the mean motion-corrected functional EPI volume, and then normalizing this co-registered volume to the Montreal Neurological Institute (MNI) EPI template volume. For the connectivity analysis, the resulting normalization parameters were applied to the functional EPI volumes. For multivariate analyses, the normalization parameters were applied to brain maps of decoder classification accuracy. In both cases, the resulting normalized volumes were spatially smoothed with a 6 × 6 × 6-mm full-width half-maximum Gaussian kernel before group-level statistical testing.

### Searchlight decoding analysis

We implemented a searchlight pattern analysis approach to identify brain regions that encoded various aspects of the CS^29^. For this method, we first specified separate GLMs using motion-corrected EPI volumes for each subject and fMRI run (five runs pre-conditioning and five runs post conditioning). Each GLM included five event-related regressors specified by the visually cued onset of inhalation for each of the four odour CS's and odourless air, and six nuisance regressors corresponding to parameters from the motion-correction step. Because there were main effects of category (minty/citrus) and session (pre-/post-conditioning) for trial-by-trial sniff peak amplitude and sniff volume (Supplementary Fig. 4), we also included a nuisance regressor consisting of the down-sampled time course of recorded sniff activity, as has been done in other studies attempting to remove noise related to physiological processes^70^.

Spatial patterns of parameters estimated from these run-wise GLMs corresponding to the different CS's were then subjected to linear support vector machine classification using the LIBSVM implementation (http://www.csie.ntu.edu.tw/~cjlin/libsvm/). For value cross-decoding (Fig. 3) using data from each 146-voxel searchlight sphere the classifier was trained on CS^+^ versus CS^−^ patterns from one category (for example, minty), and then tested on CS^+^ versus CS^−^ patterns from the ‘left-out' category (for example, citrus). The procedure was then repeated with the training and test designations switched, and the average accuracy of the two iterations was mapped to the centre voxel of the sphere. This cross-decoding technique ensures that above-chance classification is not confounded by category differences, and tests specifically for value-related encoding that generalizes across perceptual category. For the category cross-decoding (Fig. 4) the classifier was trained on mCS^+^ versus cCS^+^ patterns and then tested on mCS^−^ versus cCS^−^ patterns (and vice versa). Here this technique ensures that decoding is not confounded by value differences across categories, and that identified brain regions contain truly category and not merely identity information. For the test of identity-based decoding (Fig. 5) we trained a classifier on patterns from all four CS simultaneously from a subset of fMRI runs (for example, runs 1–9) and then tested the classifier on patterns from the left-out run. This process was cross-validated such that each run served as the left-out run in an iteration.

For all three types of classifiers, we performed the decoding analysis separately in the pre- and post-conditioning data sets. Accuracy maps resulting from these analyses were normalized and smoothed as described above, and submitted to group-level paired *t*-tests to test for learning-related changes in neural encoding. Because we did not observe changes in perceptual category (Fig. 4) or identity (Fig. 5) decoding, we performed additional searchlight analyses for these two decoders using data from both pre- and post-conditioning sessions combined.

### PPI analysis

We used the generalized form of the psycho-physiological interaction (PPI) model^31^,^67^ to separately test for learning-related changes in connectivity between the PC (that is, category-coding region) and OFC (that is, stimulus-coding region) and the rest of the brain. For both analyses the seed region was defined by the group-level analysis using combined pre- and post-conditioning runs, with threshold set to *P*<0.001 uncorrected. For each seed region analysis, we first specified PPI models at the subject level using normalized, smoothed functional EPI images as the data, condition-specific inhalation onset times for each run as the ‘psychological' factor and the first eigenvariate of seed region activation as the ‘physiological' factor. These models also included nuisance regressors for the run-wise sniff traces and motion-correction parameters, as was done for the GLMs used in the searchlight pattern analysis described above. Estimated connectivity parameters corresponding to each CS and session were then entered into group-level one-way ANOVAs, which were then tested for a main effect of session (that is, post-conditioning>pre-conditioning).

### Statistical analysis

Learning-related changes in classification accuracy were tested at the group level using paired *t*-tests (that is, post-conditioning>pre-conditioning). In the case of analyses using data from pre- and post-conditioning sessions combined, significance was tested at the group level using one-sample *t*-tests on classification accuracy. Because subjects required varying numbers of trial blocks during the conditioning phase to reach criterion, all group-level models included the total number of conditioning blocks as a nuisance covariate. Significance was tested at the group level in an explicit mask consisting of regions known to be involved in odour perception and reward processing, including hippocampus, amygdala, PC, striatum, anterior insula, OFC and anterior cingulate cortex. Significance at the group level was set at *P*<0.05, small volume corrected for multiple comparisons (family-wise error rate, FWE) using corresponding anatomical regions defined by the automated anatomical labelling atlas. For display purposes, voxels that survive uncorrected thresholding at *P*<0.001 are shown in red, and voxels that survive FWE small volume correction at *P*<0.05 are shown in yellow.

## Additional information

**How to cite this article:** Howard, J. D. *et al*. Converging prefrontal pathways support associative and perceptual features of conditioned stimuli. *Nat. Commun.* 7:11546 doi: 10.1038/ncomms11546 (2016).

## Supplementary Material

Supplementary Information Supplementary Figures 1-4 and Supplementary Tables 1-2
