# Supplementary material for: Converging prefrontal pathways support associative and perceptual features of conditioned stimuli
Source: Nat Commun. 2016 May 4;7:11546. doi: 10.1038/ncomms11546 (PMC4857483; doi:10.1038/ncomms11546)
Supplement: Supplementary Information — Supplementary Figures 1-4 and Supplementary Tables 1-2 [file ncomms11546-s1.pdf]

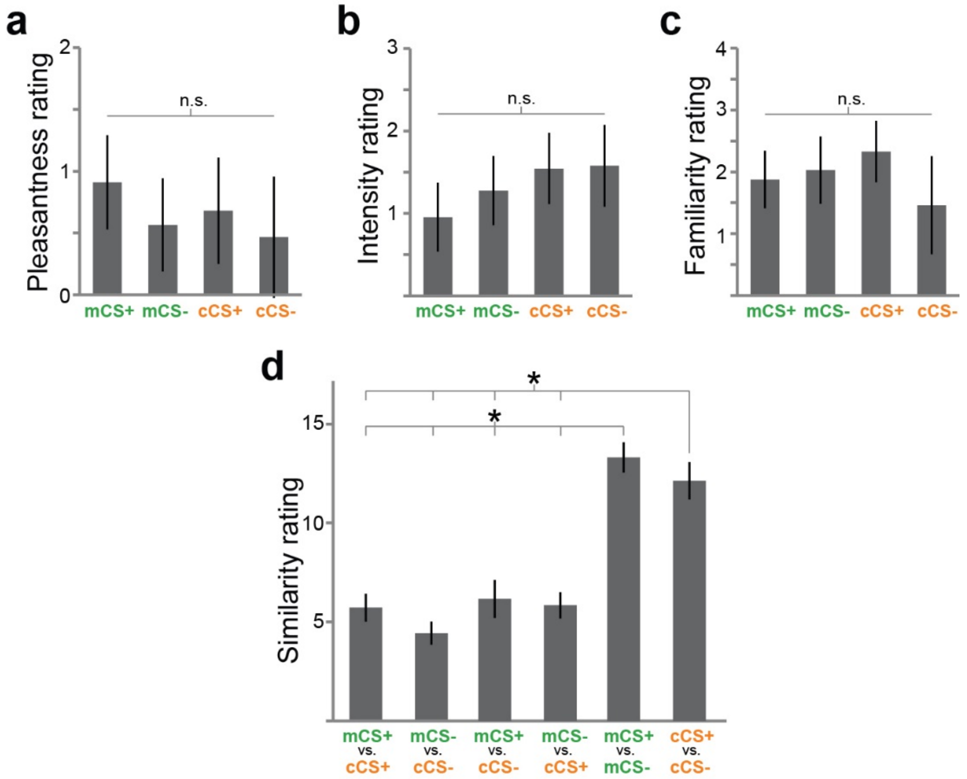

Supplementary Figure 1: Screening session behavioral ratings
In order to establish an odor stimulus set for each subject, we conducted an initial behavioral testing session approximately 7 days prior to the main fMRI scanning experimental session (in the same subjects). There were no differences in (**a**) pleasantness (main effect of category, $F_{1,14} = 0.23$, $P = 0.64$; main effect of reward, $F_{1,14} = 0.86$, $P = 0.37$; category x reward interaction, $F_{1,14} = 0.10$, $P = 0.75$), (**b**) intensity (main effect of category $F_{1,14} = 4.10$, $P = 0.072$; main effect of reward, $F_{1,14} = 0.17$, $P = 0.69$; category x reward interaction, $F_{1,14} = 0.25$, $P = 0.63$), or (**c**) familiarity ratings (main effect of category $F_{1,14} = 0.01$, $P = 0.93$; main effect of reward, $F_{1,14} = 0.34$, $P = 0.57$; category x reward interaction, $F_{1,14} = 1.60$, $P = 0.23$) for the four chosen odors, binned according to their to-be-rewarded schedules. (**d**) Within-category ratings of pairwise odor similarity were significantly greater than across-category ratings (all P's < 0.001, paired t-tests on each within-category pair vs. each across-category pair), and there was no difference between within-category minty ratings and within-category citrus ratings ($t_{14} = 1.30$, $P = 0.22$). Together these results suggest that the four odors belonged to two distinct perceptual categories. Note, mCS^+ and cCS^+ refer to the minty and citrus odors randomly chosen to be paired with $1.00 rewards in the conditioning session of the main experiment. mCS^- and cCS^- were paired with no reward. Error bars represent within-subject s.e.m.

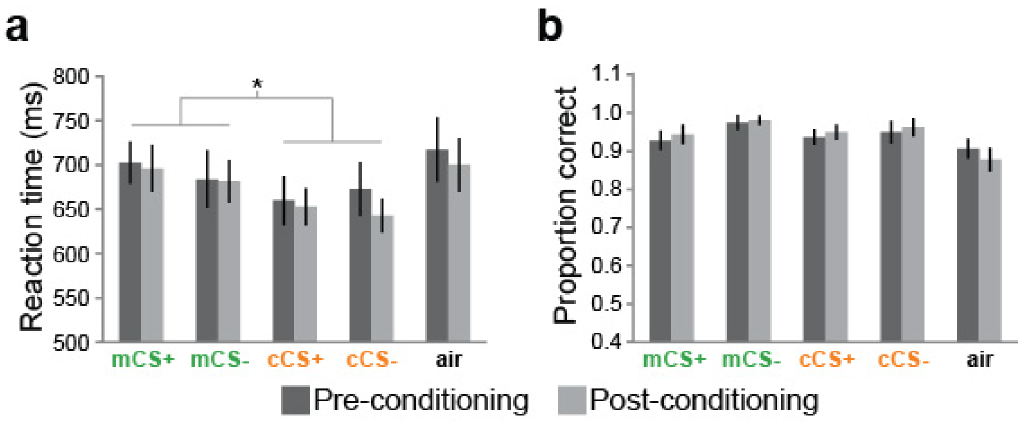

Supplementary Figure 2: Reaction times and performance accuracy on the within-scanner odor detection task
(a) The time taken to make either a yes or no response on the odor detection task performed in the fMRI scanner was calculated relative to the time the sniff cue was presented. These times were sorted by odor condition and averaged across trials within each testing session. In a 3-way ANOVA with reward outcome, session (pre- vs. post-conditioning), and perceptual category as factors, we found a main effect of perceptual category ($F_{1,14}$ = 10.2, P = 0.0065), such that subjects responded faster to the citrus than the minty odors. This may have been related to the non-significant trend towards the citrus odors being perceived as more intense than the minty odors (see **Supplementary Figure 1b**). However, there was no significant interaction between session and reward outcome (P = 0.54), suggesting that this effect was unrelated to the change in value demonstrated for the CS^+ odors. **(b)** The proportion of correct responses in the odor detection task performed in the fMRI scanner was sorted by condition and averaged across trials within each testing session. We found no significant main effects or interactions in a 3-way ANOVA with reward outcome, session, and category as factors (P's > 0.27). Reaction times and task performance for the air condition are shown here but were not included in these analyses. Error bars represent within-subject s.e.m.

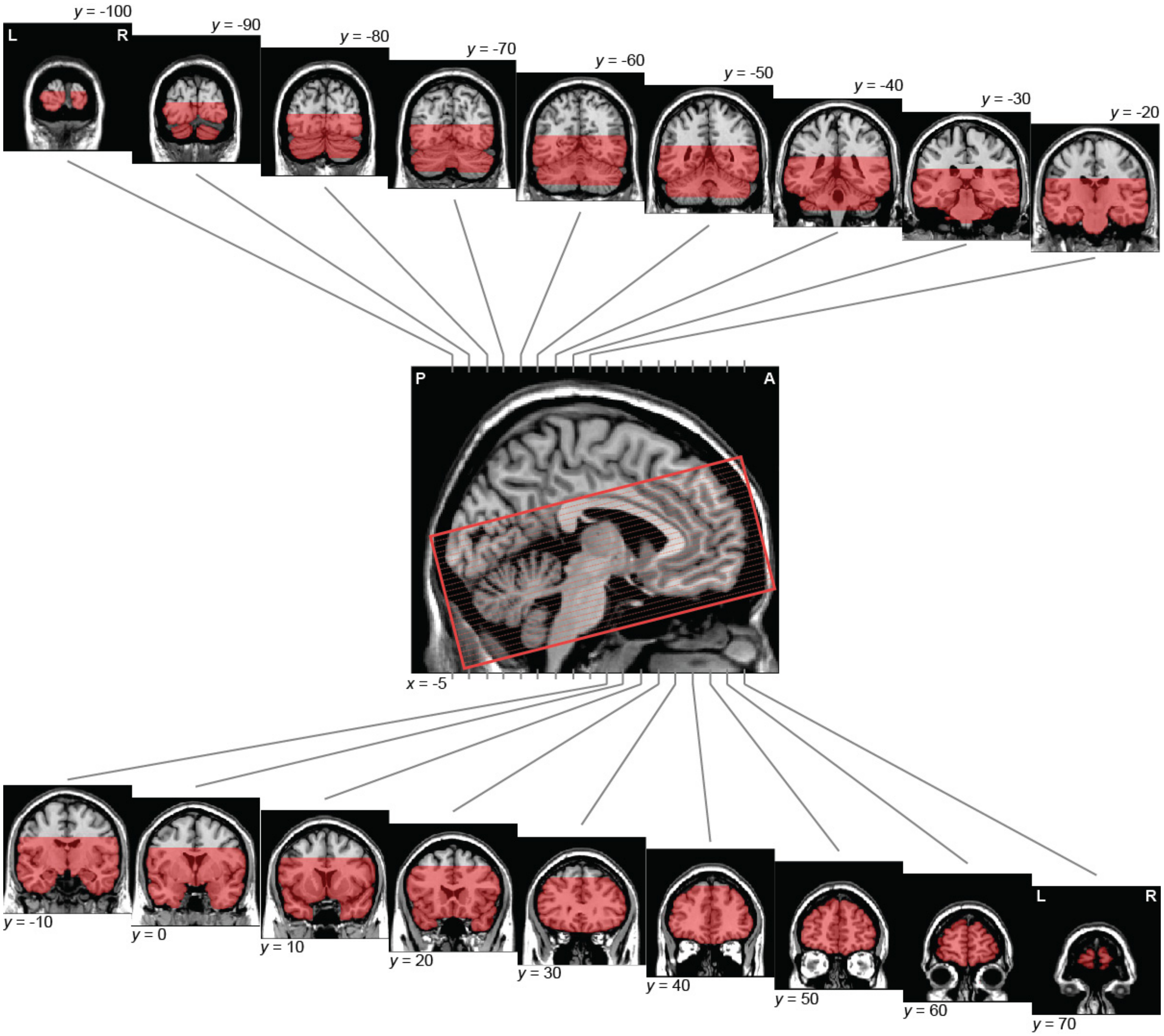

Supplementary Figure 3: fMRI scanning coverage
The imaging window of our 24-slice functional EPI sequence, shown in the sagittal view in the center of the figure, is tilted relative to the anterior commissure/posterior commissure line to minimize signal artifact in orbitofrontal and medial temporal regions. Red shaded regions in the coronal plane slices demonstrate the typical extent of the coverage resulting from this tilt on an example brain. Coordinates refer to MNI space.

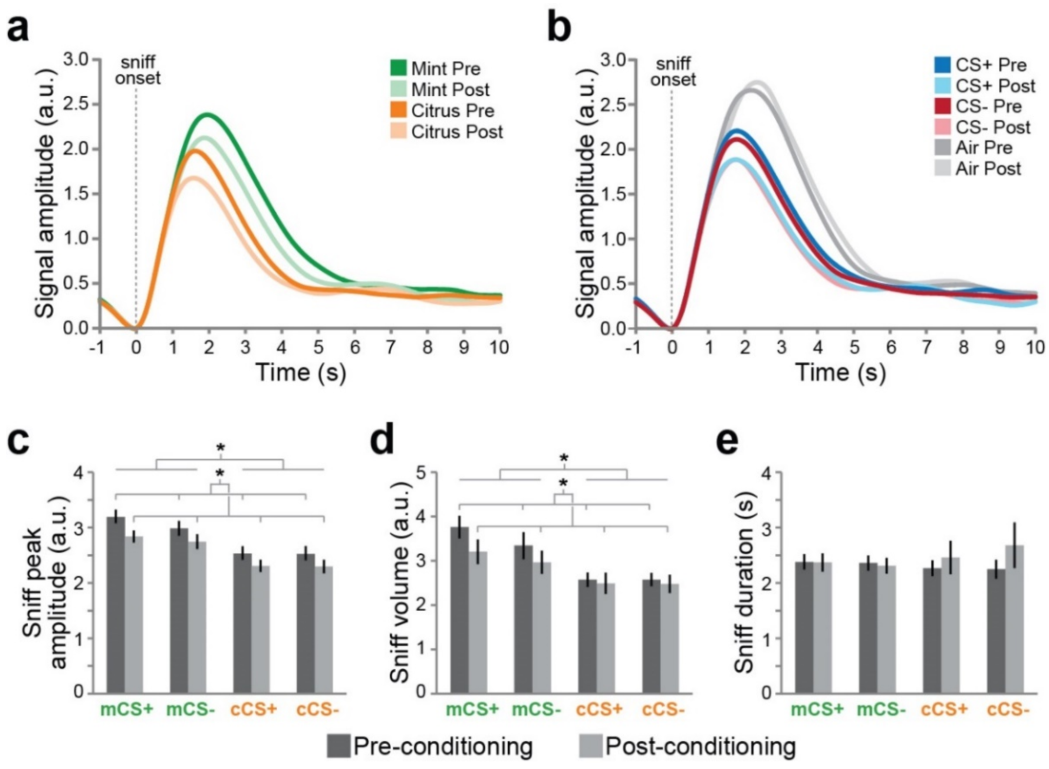

Supplementary Figure 4: Odor detection task sniff analysis
Sniff data acquired during each fMRI run were normalized, and trial-specific sniff traces were extracted and sorted by condition. For each trial, the onset of the sniff onset was determined by the signal minimum in a time window spanning 1s before and after the participant was cued to make a sniff. Each trial-specific sniff trace was then lined up according to this onset time. (**a**) Trial-specific sniff traces sorted by odor quality and session, and averaged across trials and subjects. (**b**) Trial-specific sniff traces sorted by reward schedule and session, and averaged across trials and subjects. For each trial-specific trace we calculated the peak amplitude, volume, and duration of the sniff. Analysis of these sniff parameters revealed a main effect of session and category for (**c**) sniff amplitude (session: $F_{1,14}$ = 16.1, P = 0.0013; category: $F_{1,14}$ = 13.8, P = 0.0023), and (**d**) sniff volume (session: $F_{1,14}$ = 16.1, p = 0.0013; category: $F_{1,14}$ = 13.8, P = 0.0023), but importantly no interactions of session x category (amplitude: $F_{1,14}$ = 0.610, P = 0.448; volume: $F_{1,14}$ = 2.53, P = 0.134). (**e**) There were no significant effects for sniff duration (*P*'s > 0.210 for all main effects and interactions). Error bars represent within-subject s.e.m.

odorant name	odorized air flow rate (L/min)	odorless air flow rate (L/min)	total low rate (L/min)
(-)-isopulegol	3.04	0.16	3.20
(*R*)-(-)-carvone	1.92	1.28	3.20
methyl salicylate	2.88	0.32	3.20
eucalyptol	0.96	2.24	3.20
citral	1.60	1.60	3.20
(*R*)-(+)-limonene	1.60	1.60	3.20
citronellyl acetate	1.60	1.60	3.20
nonanal	1.60	1.60	3.20

Supplementary Table 1 – Flow rates used for each odorant. Total flow was held constant at 3.20 L/min throughout the experiment.

	(-)-isopulegol	(*R*)-(-)-carvone	methyl salicylate	eucalyptol	citral	(*R*)-(+)-limonene	citronellyl acetate	nonanal
sub1	+	−			−	+		
sub2	−		+			−	+	
sub3	+				+		−	
sub4	−		+		−			+
sub5	−		+				+	−
sub6		+	−		+	−		
sub7	−	+			−	+		
sub8	−	+				+	−	
sub9		+		−		+	−	
sub10	+		−				+	−
sub11	+			−			−	+
sub12		−		+			+	−
sub13	+	−			+		−	
sub14		−	+		−	+		
sub15		+		−	+	−		

Supplementary Table 2 – Odors selected and randomized conditioning schedule (plus sign = CS^+, minus sign = CS^-) for each study participant.
